# A Comparison among Different Strategies to Detect Potential Unstable Behaviors in Postural Sway

**DOI:** 10.3390/s22197106

**Published:** 2022-09-20

**Authors:** Bruno Andò, Salvatore Baglio, Salvatore Graziani, Vincenzo Marletta, Valeria Dibilio, Giovanni Mostile, Mario Zappia

**Affiliations:** 1 Department of Electric Electronic and Information Engineering, DIEEI, University of Catania, 95125 Catania, Italy; 2 Department of Medical, Surgical Sciences and Advanced Technologies “GF Ingrassia”, University of Catania, 95100 Catania, Italy; 3 Oasi Research Institute—IRCCS, 94018 Troina, Italy

**Keywords:** postural stability, wearable devices, accelerometer, threshold algorithm, Neuro-Fuzzy inference, Discrete Wavelet Transform

## Abstract

Assistive Technology helps to assess the daily living and safety of frail people, with particular regards to the detection and prevention of falls. In this paper, a comparison is provided among different strategies to analyze postural sway, with the aim of detecting unstable postural status in standing condition as precursors of potential falls. Three approaches are considered: (i) a time-based features threshold algorithm, (ii) a time-based features Neuro-Fuzzy inference system, and (iii) a Neuro-Fuzzy inference fed by Discrete-Wavelet-Transform-based features. The analysis was performed across a wide dataset and exploited performance indexes aimed at assessing the accuracy and the reliability of predictions provided by the above-mentioned strategies. The results obtained demonstrate valuable performances of the three considered strategies in correctly distinguishing among stable and unstable postural status. However, the analysis of robustness against noisy data highlights better performance of Neuro-Fuzzy inference systems with respect to the threshold-based algorithm.

## 1. Introduction

An Active and Healthy Ageing (AHA) society would be a resource from which everyone can benefit and the development of an AHA scenario is gaining more and more interest all over the world [1]. Maintaining a healthy ageing population may lower the demands for health care services, while being supportive to their fellow generation. In order to face this challenge, several initiatives have been launched, promoting worldwide active and healthy ageing. From a public health perspective, frailty is a multidimensional issue resulting from changes in physical and mental health and functional status, as well as lack of social and economic resources. Functional decline is often associated with lower psychosocial status, namely social isolation, malnutrition, and comorbidity, which are all determinants of frailty. Falls and their predictive behaviors, such as postural instabilities, are major contributors to functional decline. They both contribute in a substantial way to the limitation of mobility and premature hospitalization. The elderly are not the only subjects affected by falls and postural instabilities. People suffering from neurodegenerative diseases, such as the Parkinson’s Disease (PD), also suffer from postural instabilities and consequent falls, often recurrent, with an estimated rate of falls per recurrent faller per year ranging from 4.7 to 67.6 within one year from the onset of the disease [2]. A range of fall prevention interventions have been developed within both research and practice. These include clinical assessment and treatment of fall risk factors, such as exercise programs that focus on balance and muscle strength, medication management, and vision checking. Although impressive progress has been made through the years, falls, and especially their prediction, still remain serious issues nowadays. In this context, the development of technologies aimed to predict suspect postural behaviors plays a fundamental role. 

In the following, a brief review of the state of the art, addressing the development of solutions for postural sway monitoring, is given. The very first classification key among different solutions already available or under development consists of the two main classes: infrastructure-dependent devices and wearable systems [3,4,5,6]. The actual approaches adopted in diagnostic premises exploit force platforms and/or vision systems [3,4]. In [3], a force plate and inertial sensor are suggested to monitor the center of pressure (COP) and multiscale entropy was used to detect fractal correlations and assess data complexity. Analysis of the main features to assess postural stability, including COP displacement, equilibrium score, and other postural stability indexes are discussed in [5], along with the equipment (force plate, balance master, and equitest) adopted to achieve above information.

Recent works investigated the possibility to use a Kinect vision system to study postural stability features or to localize the center of mass (CoM) of an upright person [5,6]. Although very accurate, the main limit of such infrastructure-dependent solutions is represented by the need to perform periodic laboratory tests, which lead to a discontinuous monitoring of the patient. With the aim of continuously monitoring potential postural instability, a different approach is required. As an example, a wearable device could be used to collect information useful for analyzing the postural sway. Among wearable systems, the most popular devices are instrumented insoles [7] and inertial-sensor-based systems [8,9,10]. Insoles represent a valuable solution for the monitoring of the Centre of Pressure (CoP), although not always easy to use and to maintain [6,7]. 

Wearable IMU-based solutions are nowadays recognized as reference tools for the monitoring of postural sway [11]. A review of the main approaches using IMU is presented in [12]. A solution based on an inertial sensor positioned on the posterior trunk at the level of the L5 lumbar vertebra is discussed in [13], with a specific focus on the system validation against reference tools (e.g., a force plate). An approach based on a tri-axial accelerometer to quantitatively classify PD patients into motor subtypes is investigated in [14]. A head-mounted wearable IMU and different proposed measures of postural sway are proposed in [15]. A recent work aimed to assess the validity of a solution based on the use of an IMU sensor in a pendant worn around the neck [16]. Other approaches also exploit the use of tablet- or smartphone-based solutions [17].

Several efforts have been performed to extract metrics useful for the qualification and quantification of postural behaviors from signals provided by sensors. Both time-based and frequency-based features have been investigated as strategic piece of information allowing for the assessment of postural behaviors [18,19,20], differentiating solutions into two further approaches. Time-based features directly estimated by tri-axial inertial sensors have been widely used in the literature [19,20]. A microcontroller-based wearable device aiming to provide the angular position in eight different locations on the user is discussed in [21]. The use of an IMU and dedicated signal processing to analyze postural status in Alzheimer patients is presented in [9]. In [22], features extracted by stabilograms were used to implement a postural sway assessment strategy. The work exploited a threshold algorithm to distinguish among stable and unstable postural behaviors. The main limitation of this approach is related to the difficulty of finding a robust element of separation among different classes of postural behaviors, which may affect the assessment reliability.

Despite the good performance of time-based approaches, the exploitation of the frequency content of inertial dynamics is getting more and more interest among researchers [23,24]. This is justified due to the understanding that increased postural movements do not necessarily indicate destabilization. Indeed, since pieces of information on postural sway are hidden in different timescales, the Discrete Wavelet Transform (DWT) could represent an effective tool to extract useful metrics from such dynamics, allowing for detection of potential unstable behaviors [25,26,27]. 

In [28], the DWT theory was exploited to detect the Critical Point Interval (CPI), which indicates a variation of the user’s postural control strategy. In [29], a deep analysis is elaborated with the aim to provide new insights into postural control by exploiting DWT to extract strategic timescale components from the center-of-pressure time series. It is clearly remarked that wavelet decomposition could be a useful tool in the analysis of postural stability because several neuromuscular feedback loops act at different discrete timescales, which could not be always present in the entire time series. Moreover, the same work offers a very well-elaborated background on wavelet transform analysis tailored to specific cases of use. In [30], DWT is used to process signals provided by a micro-controlled-based inertial wearable system with the aim of extracting useful information related to postural sway behavior.

A further key classification point is represented by the kind of paradigm adopted to process features extracted by signals provided by sensors, both in the time and frequency domains. Two major classes are represented by CRISP and Machine Learning (ML) approaches. In [30], the authors proposed a methodology for the postural-instability-detection-exploiting features provided by the DWT theory and a threshold-based classification strategy. In [22], features extracted by the time evolution of Antero-Posterior and Medio-Lateral Displacements were used with a threshold-based paradigm to detect potential instability in the postural behavior. ROC curves theory was used to estimate the optimal thresholds for each adopted feature. Although high sensibility of the methodology was demonstrated in distinguishing among stable and unstable behaviors, the reliability associated to the assessment of each postural dynamic was not always well performing. Machine-learning-based approaches were also efficiently exploited for the development of assistive solutions in fall detection [31,32,33]. In [34], an ML-based solution for the monitoring of the pressure and sitting posture prediction is presented. The paper compares decision tree classifiers against random forest.

A machine learning approach to assess the accuracy and feature importance of various postural sway metrics to differentiate individuals with Multiple Sclerosis from healthy controls, as a function of physiological fall risk, is presented in [35]. A random forest algorithm was used to predict individuals’ fall risk grouping. A machine learning model to classify the severity of the motor part of PD patients from their gait, monitored by smartphone sensors, is presented in [36].

Among other techniques, the use of Neuro-Fuzzy paradigms in the field of postural sway assessment is well documented in the literature. As an example, in [37], the authors investigate the associations between gait performance, postural stability, and depression in patients with PD by using an adaptive NF inference system. In [38], a Fuzzy Logic classification algorithm aimed to classify the quality of postures using information gathered by the center of pressure, the Posture Adoption Time, and other related features. In [39], a Neuro-Fuzzy approach was used to classify among stable and unstable behaviors. The obtained results demonstrated the suitability of the proposed methodology against threshold-based approaches.

This paper aims to offer a comparison among different approaches to detect potential postural instability from the stabilogram, achieved by using a wearable inertial system. In particular, the considered methodologies include a threshold-based algorithm and an NF model, both processing time-based features, as well as an NF inference system using frequency-based features obtained by a DWT operator. The latter, which represents the main new contribution introduced by this work, is worthy of investigation in order to assess the performance of a combined approach using DWT-based metrics and an NF algorithm.

The main outcomes of this work may be summarized as follow:

1. Performing a comparison among different strategies for postural sway analysis, aiming to distinguish among stable and potential instable postural behaviors;
2. Stimulating the idea of using a combined threshold-based and NF approaches to detect potential postural instabilities, considering limitations given by the low reliability of the former and difficulty of implementation in real-time embedded nodes of the latter, as discussed in Section 4;
3. The definition of metrics for the assessment of the proposed methodology;
4. The definition of a performance index rating the reliability of the postural sway detection;
5. Testing the new approach of using DWT to extract suitable features representing the postural sway dynamics in combination with an NF strategy to implement a methodology for postural instability detection;
6. The use of a wider dataset, with respect to [22,39], allowing for the performance of a better assessment of the different proposed methodologies;
7. The analysis of robustness against noisy data, which demonstrates that the NF approach performs better than threshold-based algorithms, especially in the presence of noisy data.

## 2. The Sensing Platform, the Experimental Set-Up, and the Adopted Dataset

The sensing node adopted to perform the experimental survey and to build the postural sway dataset consists of an STM32 platform, exploiting an ultra-low-power ARM Cortex-M4 microcontroller with DSP and the LIS2DW12 MEMS accelerometer, by STMicroelectronics. The sensor is a 16-bit ultra-low-power three-axis linear accelerometer, with output data rates from 1.6 Hz to 1600 Hz and selectable full scales of ±2 g/±4 g/±8 g/±16 g [40]. A sampling rate of 100 Hz was used in line with approaches suggested in the literature. Acquired data were then stored in a SD card.

**Figure 1:**
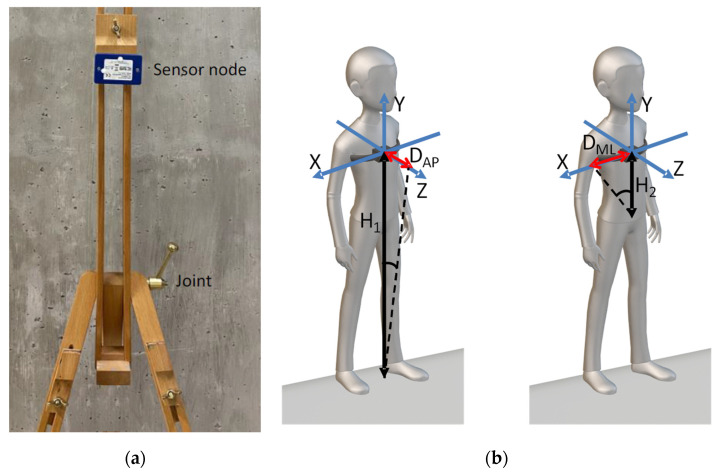
(**a**) The architecture miming standing postural sway behaviors; (**b**) the equivalent node position and representation of main quantities useful for reconstructing the AP and ML dynamics.

The sensor node was mounted on a dedicated structure, shown in Figure 1a, allowing for miming Stable behaviors (ST), Antero-Posterior (AP) and Medio-Lateral (ML) dynamics, and overall instable behaviors (INST). The node was positioned at a height from the floor corresponding to standard chest positions. This choice was strictly related to the need of acquiring a consistent dataset of different standing postural behaviors without the need, during this development phase, of annoying real patients.

The structure was moved manually to mimic different postural behaviors. Tilting movements around the belt joint allowed for emulating Medio-Lateral displacement, while Antero-Posterior dynamics were reproduced by tilting movements around the bottom joint. For all above cases, over-threshold displacements (in one direction or AP-ML combinations) defined instable behaviors, while minor displacements were considered as stable dynamics.

Of course, while this approach could be suitable during the development phase due to reasons above mentioned, its main limitations are given by artefacts introduced by the rigid structure against human body dynamics. However, this should not compromise the validity of the developed approaches, which are robust against patterns’ modifications, as also demonstrated by the analysis of the postural detection methodology against noisy data.

It must be underlined that the main aims of this paper are to develop methodologies for identifying potential postural instability, the definition of the approach (from patterns acquisition to features extraction and postural instability detection), and the comparison among different solutions.

Future efforts will be dedicated to assessing the whole approach by exploiting patterns acquired by end-users. Of course, due to different effects which will be introduced by human body shape, the main parameters of postural detection algorithms will have to be re-estimated.

The dataset composition is shown in Table 1, where 6 different cases were considered by varying the heights *H*_1_ and *H*_2_ shown in Figure 1b. Each pattern was tagged by binary information: 0 codifying stable behaviors and 1 for unstable cases (used for the estimation and the assessment of the postural detection algorithm). The dataset was then randomized and divided into a training set and a test set (ratio: 60%/40%).

The device allows for performing a continuous acquisition and data processing on time windows of 10 s shifted by 1 s to each other, as suggested in the literature [41]. A study was performed to define the windows overlap by observing the system behavior for various time shifts belonging to [0.25, 2] s. The choice of a 1 s time shift among consecutive windows was considered as a good compromise between the need for continuous postural sway monitoring and the required computational power.

## 3. Methods

In the following, different approaches for postural sway analysis are discussed. As a general remark, it must be considered that the system uses inertial data provided by a dedicated sensor node to reconstruct the movements of the user’s trunk in the two AP and ML directions. After the extraction of the acceleration components and pre-filtering, the two main Displacements, Antero-Posterior (DAP) and Medio-Lateral (DML), are computed and used to reconstruct trajectories (stabilogram) of the sensor node’s center of mass:

(1) $$\mathrm{DAP}\;=\;H_{1}\frac{A_{z}}{\sqrt{A_{y}{}^{2}+A_{x}{}^{2}}}$$

(2) $$\mathrm{DML}\;=\;H_{2}\frac{A_{x}}{\sqrt{A_{y}{}^{2}+A_{z}{}^{2}}}$$

where *H*_1_ and *H*_2_ are defined in Figure 1b, while *A_x,y,z_* are the acceleration components.

It is mandatory to highlight that the postural sway status has to be assessed in standing conditions, and hence dynamics introduced by daily activities performed by the user, such as walking, must be detected and not considered as useful conditions to assess the postural behavior [22]. To assess the postural behavior, different features can be extracted from the stabilograms. The main features considered in the present work are summarized in Table 2 [19,20]. The above features were selected, according to the literature, as belonging to the set of best features to be used for the aim of postural analysis. Moreover, such quantities, as defined in Table 2, are easy to be implemented on embedded systems for real-time computation.

In the following, a brief introduction is dedicated to threshold-based and Neuro-Fuzzy approaches exploiting time domain features, which were extensively presented in [22,39]. A specific focus is then dedicated to the NF inference system processing DWT-based features, which has not been addressed up to now, thus representing a new contribution by this work. The results obtained by processing the dataset introduced in Section 2 are presented and discussed in Section 4.

### 3.1. The Time-Features-Based Threshold Algorithm

**Figure 2:**
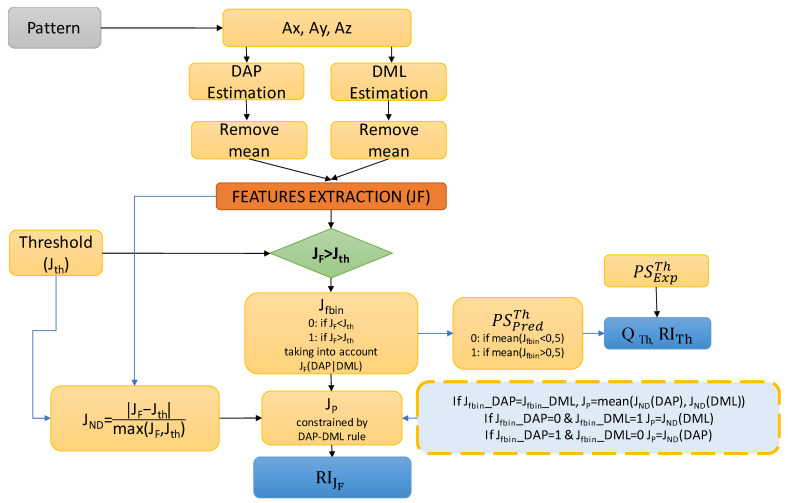
The threshold algorithm considered in this work to analyze the postural sway and to detect potential unstable behaviors [22].

The threshold algorithm considered in this work to analyze the postural sway and to detect potential unstable behaviors is deeply discussed in [22]. For the sake of convenience, in the following, the adopted approach, represented in Figure 2, is briefly summarized. The threshold algorithm is mainly based on the comparison between the values of each feature (ref. Table 2), *J_F,_* and the corresponding threshold, *J_th_*. Thresholds were estimated by using the first part of the dataset and exploiting the Receiver Operating Characteristic (ROC) theory [22]. The result of this comparison is a set of binary features, *J_fbin_*, which can assume the value 0 in case the feature is under-threshold; otherwise they are 1. Considering the strong specificity of features $D_{AP,\;ML}^{max}$ against unstable behaviors along the AP and ML directions, the corresponding *J_fbin_* indexes are combined by a logic OR operator. In case at least 50% of considered features shows values higher than the corresponding thresholds, the observed dynamic is classified as potentially unstable.

In order to assess the performance of the threshold-based approach, the following index was defined:

(3) $$Q_{Th}\%=100\left[1-\frac{\sum_{i=1}^{N}\left|PS_{Pred}^{Th}-PS_{Exp}^{Th}\right|}{N}\right]$$

where:

− $PS_{Pred,\;Exp}^{Th}$ are the predicted and expected postural behavior;
− *N* is the number of the considered patterns.

It is also mandatory to define a reliability index, which allows for assessing the quality of the postural status predicted by the algorithm.

To such an aim, the following quantity was defined, which weights the distance between the mean value of *J_fbin_*, estimated across the whole set of considered features, and the separator element (0.5) between the two classes of postural sway (stable (0), unstable (1)):

(4) $$RI_{Th}\%=100\;\left[\frac{\left|\frac{1}{N_{F}}\sum_{q=1}^{N_{F}}J_{fbin}-0.5\right|}{0.5}\right]$$

where *N_F_* is the number of considered features.

For the sake of completeness with the approach proposed in the previous work [22], the following quantity was also used, which weights the average distance among values of each feature and the corresponding threshold:

(5) $$RI_{J_{F}}\%=100\left(\frac{1}{N_{F}}\overset{N_{F}}{\underset{q=1}{\sum }}J_{P}\right)$$

where:

− *J_P_* is calculated by the operator $JND=\frac{\left|J_{F,q}-J_{th,q}\right|}{\max\left(J_{F,q}-J_{th,q}\right)}$, constrained by the rule reported in the dotted box in Figure 2, taking into account the peculiarity of $D_{AP,\;ML}^{max}$ features;
− *J_F,q_* states the value of the *q* considered features.

While index (5) is more suitable to assess the detection strategy during the development phase, index (4) represents a convenient way to assess the reliability of the predicted postural status.

### 3.2. The Time-Features-Based Neuro-Fuzzy Approach

As well depicted in [39], features do not always allow for a sharp separation between stable and unstable behaviors. In many cases the values of such features are very close to the thresholds adopted as separators among the two classes of stable and unstable behaviors. As a consequence, threshold-based approaches are prone to suffer for a low robustness against noisy data, with respect to ML approaches. In particular, this effect could mainly affect the reliability of postural status predicted by the paradigm. On the basis of this consideration, other approaches based on ML were explored to accomplish the above-mentioned tasks [39].

With the aim of comparing among different postural status detection methodologies, in this work, the Sugeno-type Fuzzy inference system presented in [39] is considered, which is particularly convenient to perform classification tasks. The initial structure was generated by using the “genfis” function in Matlab, which allows for extracting rules. The number of rules and antecedent membership functions were fixed by subtractive clustering, while each rule’s consequent equations were obtained by a linear least-square estimation. The “anfis” function was used to implement the training phase, thus generating a single-output Sugeno fuzzy inference system and tuning the system parameters to the considered dataset by least-squares and backpropagation gradient descent methods. A grid partitioning approach was then used to finalize the model.

Finally, the “evalfis” function was used to estimate the predicted Postural Status, $PS_{Pred}^{NF}$, which were then rounded to the closest 0 or 1 value, as defined by $PS_{Round}^{NF}$.

A strategic parameter to be fixed in the NF inference system is the “Range of influence of the cluster center”, which defines the range of the search for clusters in a dataset. The results allowing for defining the optimal value of the Range of Influence are presented and discussed in Section 4.

Additionally, in this case, the following quantities were used to assess the methodology and to provide a reliability index for each predicted postural status:

(6) $$Q_{NF}\%=100\left[1-\frac{\sum_{i=1}^{N}\left|PS_{Round}^{NF}-PS_{Exp}^{NF}\right|}{N}\right]$$

(7) $$RI_{NF}\%=100\left[\frac{\left|PS_{Pred}^{NF}-0.5\right|}{0.5}\right]$$

where:

− $PS_{Exp}^{NF}$ is the expected postural status and 0.5 is the separator element between the 2 classes (0—Stable, 1—Unstable).

### 3.3. The DWT-Features-Based Neuro-Fuzzy Approach

In order to implement this methodology, time variant DAP and DML signals were considered. The 5-level DWT of the DAP and DML were computed over time windows of 10 s shifted by 1 s to each other. Successively, the following features were estimated (Mean Value (MV), Standard Deviation (STD), and Energy (E), which the literature recognizes as the most meaningful, in order to accomplish the task under consideration [28,29,30].

(8) $$MV\left(a\right)=\frac{1}{K\left(a\right)}\overset{K(a)}{\underset{K=0}{\sum }}T\left(a,k\right)$$

(9) $$STD\left(a\right)=\sqrt{\frac{1}{K\left(a\right)-1}\overset{K(a)}{\underset{K=0}{\sum }}{\left(T\left(a,k\right)-MV\left(a\right)\right)}^{2}}$$

(10) $$E\left(a\right)=\overset{K(a)}{\underset{K=0}{\sum }}T{\left(a,k\right)}^{2}$$

where ‘*T*(*a*, k)’ represents a wavelet at timescale *a*, identified by levels *d1, d2, d3, d4, d5*, and *K* the number of samples in the transformed signal.

In order to reduce the number of inputs of the postural detection algorithm, the approach proposed in [30] was followed, mainly considering lower levels (*d3*, *d4*, and *d5*) of the Wavelet Coefficients (WCs). As an example, Section 4.2 shows that values of the strategic feature (8) assume greater amplitude for levels (*d3, d4*, and *d5*) than for levels (*d1* and *d2*).

**Figure 3:**
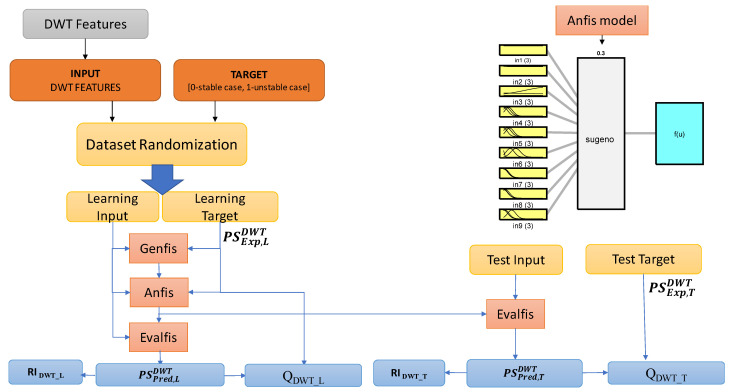
The Neuro-Fuzzy inference system considered in this work to analyze the postural sway and to detect potential unstable behaviors [39].

The above features were computed on the DAP and DML displacements, for levels *d3*, *d4*, and *d5*, and then summed together, thus producing a dataset of 9 inputs (3 features by three levels). The obtained dataset was used to feed a Sugeno-type Fuzzy inference system. Figure 3 represents the adopted dataflow for the implementation of this approach.

The tools exploited to implement this approach were the same presented in Section 3.2. The “*evalfis*” function was used to predict the Postural Status, $PS_{Pred}^{DWT}$, which was then rounded to the closest 0 or 1, $PS_{round}^{DWT}$. Other peculiarities of the inference system are similar to the structure described in Section 3.2.

The following index was used to assess the performance of the postural sway predictive model:

(11) $$Q_{DWT}\%=100\left[1-\frac{\sum_{i=1}^{N}\left|PS_{Round}^{DWT}-PS_{Exp}^{DWT}\right|}{N}\right]$$

where:

− $PS_{Exp}^{DWT}$ is the expected postural status;
− *N* is the number of the considered patterns.

In order to assess the reliability of the postural status predicted by the proposed methodology, the following index was defined:

(12) $$RI_{DWT}\%=100\left[\frac{\left|PS_{Pred}^{DWT}-0.5\right|}{0.5}\right]$$

which weights the postural status estimated by the inference system against the average separator (0.5) between the expected output values (0, 1) associated to the stable and unstable states, respectively.

## 4. Experimental Results and Discussion

In this section, the results obtained by processing the dataset illustrated in Section 2, through the three proposed methodologies discussed in Section 3, are shown. As already mentioned, the patterns were acquired by using the embedded system, equipped by a MEMS accelerometer, presented in Section 2 and positioned on a dedicated structure allowing for the reproduction of different postural dynamics with the Medio-Lateral and Antero-Posterior degrees of freedom.

In order to estimate the overall performances of the three developed algorithms, other than the quantities defined by Equations (3), (6), and (11), the following indexes were computed through the whole learning and test datasets:

(13) $$RI_{Mean}^{}\%=mean\left(RI_{m}\%\right)$$

(14) $$RI_{Std}^{}\%=Std\left(RI_{m}\%\right)$$

where:

− *mean(.)* and *Std(.)* are the average and standard deviation operators, respectively;
− *m* states the postural status detection methodology (*Th*, *NF*, *DWT*).

### 4.1. Postural Status Detection Approaches Based on Time-Based Features

**Figure 4:**
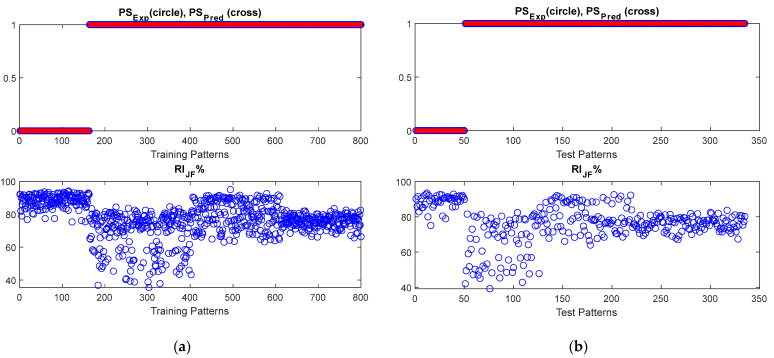
The behavior of the threshold-based algorithm for postural status detection. Both (**a**) training and (**b**) test datasets are shown.

**Figure 5:**
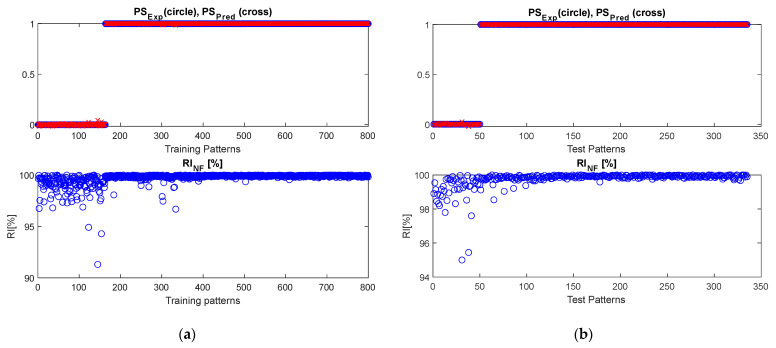
The behavior of the NF inference system for postural status detection. Both (**a**) training and (**b**) test datasets are shown.

The threshold algorithm, presented in [22] and discussed in Section 3.1, and the NF inference system, presented in [39] and discussed in Section 3.2, were fed by features presented in Table 2 and calculated over the dataset introduced in Section 2. The performances of the inference system were observed for different values of the “Range of influence of the cluster center” ranging in [0.1:0.6]. An optimal value of 0.3 was estimated for the range of influence, as it minimizes index (6). Results of both approaches are shown in Figure 4 and Figure 5, in terms of the comparison between the predicted and expected postural status and the reliability index (5), $RI_{J_{F}}$, estimated for each considered pattern.

As it can be observed, both algorithms performed very well in correctly distinguishing among stable and unstable postural status. Due to the nature of the dataset (obtained by emulating postural sway behaviors), *RI_Th_* is almost 100%, which is comparable to the *RI_NF_* obtained for NF-based approaches.

The reliability index $RI_{J_{F}}$, related to the threshold-based approach, highlights poor robustness for some of performed predictions, due the closeness of features values to the estimated thresholds. This result is further confirmed by the robustness-against-noise analysis presented in Section 4.3.

### 4.2. The Postural Status Detection Approach Based on DWT-Based Features

In this section, a special focus is dedicated to the results obtained by NF inference system fed with DWT-based features.

**Figure 6:**
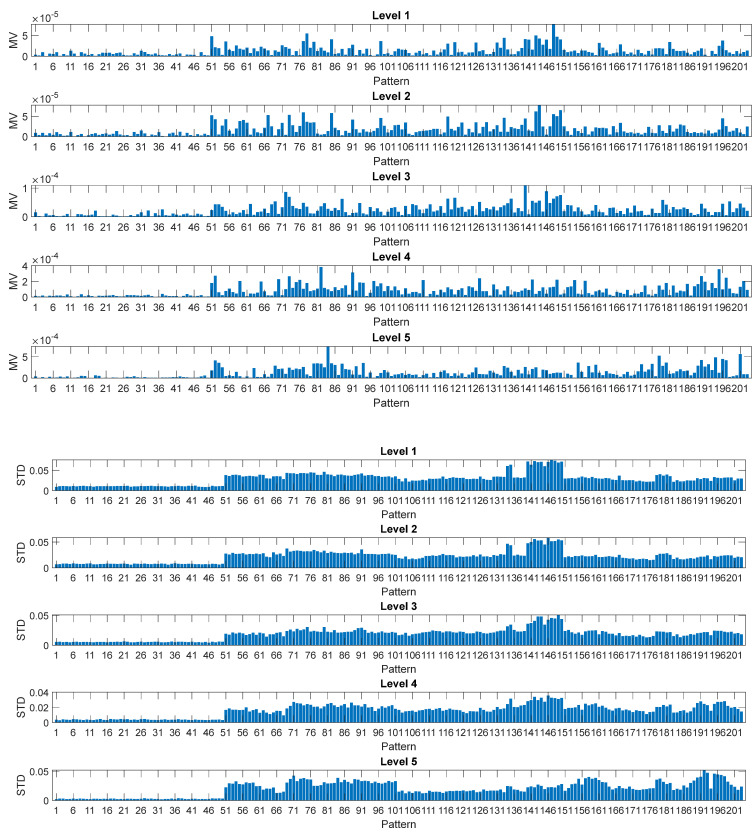

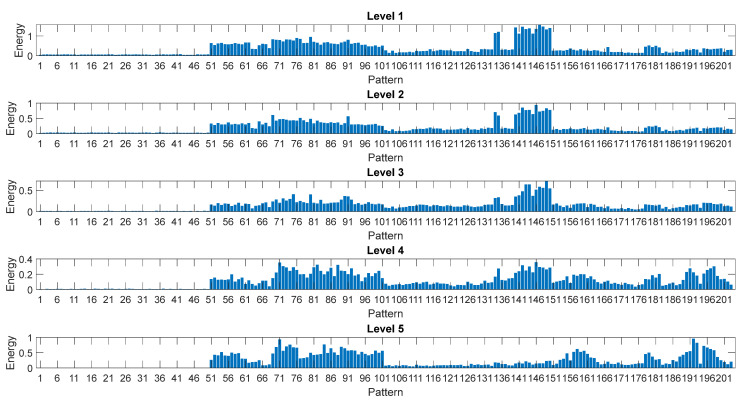
Features (8)–(10) estimated by the DWT for each of the computed levels d1-d5.

Figure 6 shows features (8)–(10) estimated by the DWT for each of the computed levels. As expected, the contribution of the first two levels is negligible and is not used to feed the NF algorithm [30]. Only a limited number of features are shown in the picture for the sake of readability.

The whole set of patterns were randomized and divided in two sub-sets used for the training and test phases (ratio 60%/40%), allowing, respectively, for estimation of the NF inference and assessment of its performance. A study was performed to estimate the best “range of influence of the cluster center” minimizing performance indexes (13) and (14).

**Figure 7:**
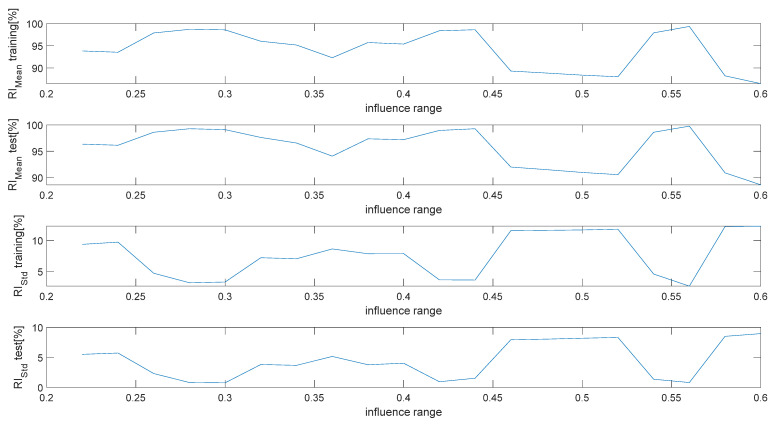
Performance indexes (12)–(13) as a function of the range of influence.

The results shown in Figure 7, representing the behavior of such quantities as a function of the range of influence, allow for fixing the latter to the optimal value of 0.3.

**Figure 8:**
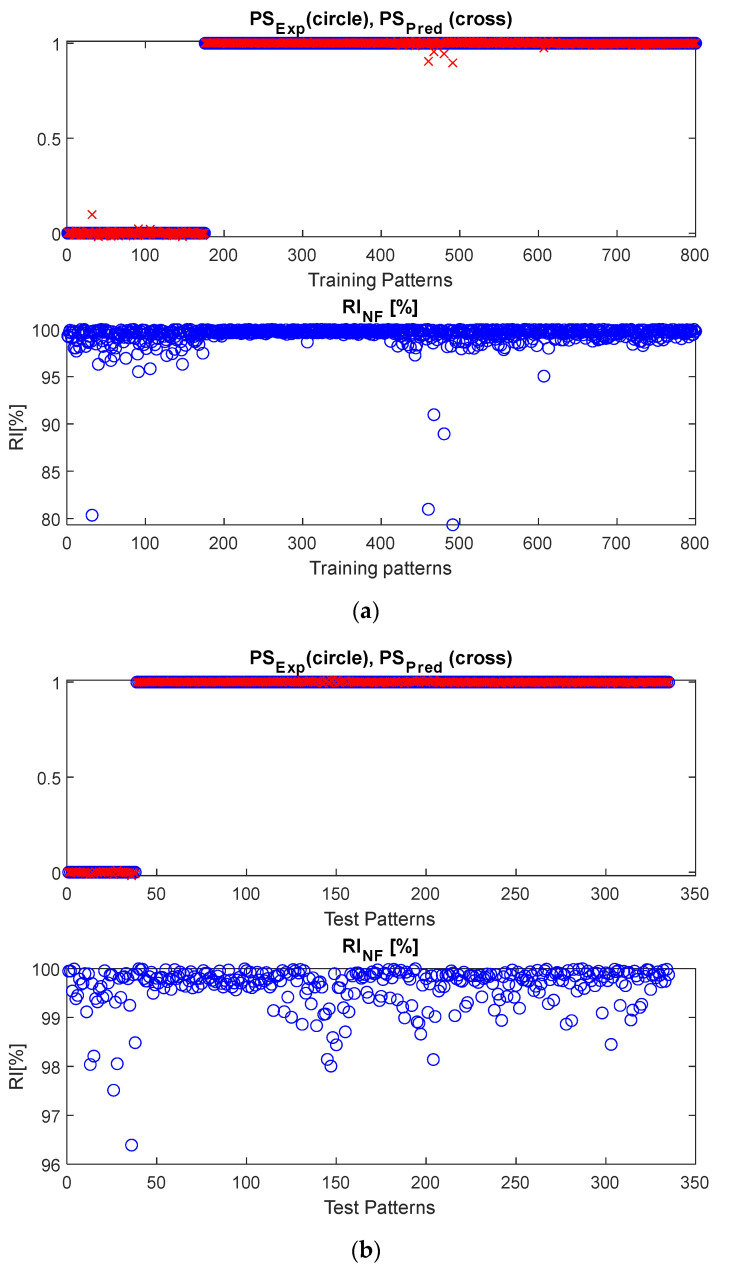
Results obtained by the NF inference system, fed with DWT-based features, in the case of the optimal range of influence. Both (**a**) training and (**b**) test datasets are shown.

Figure 8 shows the results obtained in the case of the optimal range of influence, both for the training and test datasets. In particular, the comparison between the predicted and expected postural status is shown, along with the reliability index. The results obtained demonstrate the suitable performance of the DWT-NF approach.

### 4.3. A Comparative Analysis among Different Methodologies

In this section, a discussion related to results achieved by the three postural detection strategies is reported.

A comparison among the results obtained by the three considered approaches is shown in Table 3. As demonstrated by the performance indexes *Q* (3), (6), and (11), the three approaches perform very well in performing the task of correctly distinguishing between stable and unstable postural status, as also demonstrated by the reliability indexes.

Apparently, the performance of the three methodologies are also comparable in terms of reliability, as demonstrated by quantities (13) and (14), calculated through indexes (5), (7), and (12). The results shown in the next section, analyzing the behavior against noisy data, demonstrate the advantages of NF-based approaches with respect to threshold algorithms.

For the sake of completeness, Table 3 also reports quantities (13) and (14), calculated through index *RI_JF_* (5). As already highlighted in Section 4.1, the threshold-based algorithm shows limited performance in terms of this index. The motivation for the observed behavior resides in the closeness of the features’ values to the detection thresholds adopted by the threshold-based algorithms, which could seriously compromise the correctness of the postural status estimation.

### 4.4. Analysis of Robustness against Noisy Data

The three strategies for the analysis and detection of potential unstable postural status were further investigated against a noisy dataset. To such an aim, the dataset was intentionally corrupted by Gaussian noise. In particular, an additive scheme was used to corrupt data with different levels of noise. In the following, the standard deviation of the noise added to each feature is expressed as the percentage of the maximum value of that feature calculated along the considered dataset. This choice allows for consideration of a homogenous effect of the added noise through the whole set of features.

**Figure 9:**
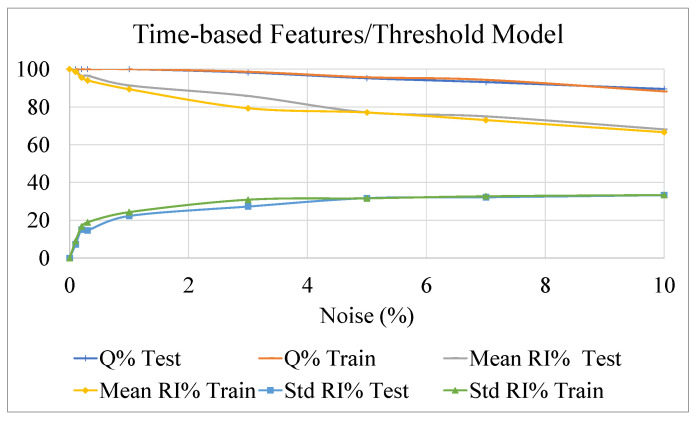
The behavior of the threshold algorithm for postural status detection, as a function of different levels of noise added to the dataset. Results for indexes (3), (13) and (14) calculated for reliability index (4) are shown.

**Figure 10:**
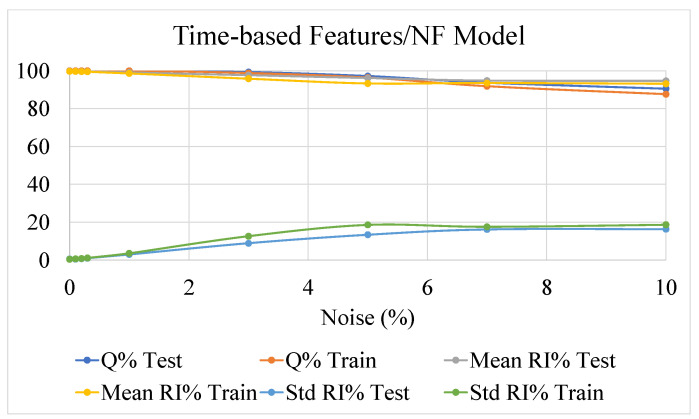
The behavior of the Neuro Fuzzy inference systems fed by time-based features, as a function of different levels of noise added to the dataset. Results for indexes (6), (13), and (14) calculated for reliability index (7) are shown.

**Figure 11:**
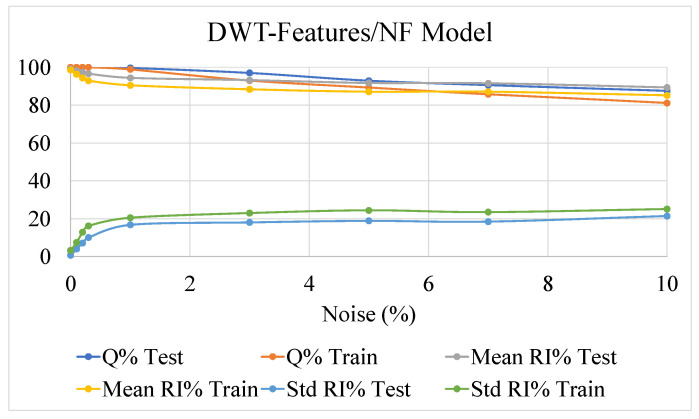
The behavior of the Neuro Fuzzy inference systems fed by DWT-based features, as a function of different levels of noise added to the dataset. Results for indexes (11), (13) and (14) calculated for reliability index (12) are shown.

The outcomes of this analysis are presented in Figure 9, Figure 10 and Figure 11, showing the effect of the noise on the accuracy indexes (3), (6), and (11) and the reliability related indexes (13) and (14), both in the learning and test datasets.

The three methodologies performed similarly in terms of the *Q%* index, assessing the robustness of such approaches in also correctly estimating the postural status in the presence of noisy data.

Concerning the reliability performance, it is worth reminding that comparison among the three methodologies must be performed by index (13) and (14), estimated through quantities (4), (7), and (12). The results obtained clearly show a remarkable robustness against noisy data of the NF-based strategies against the threshold-based approach.

The performances of the two NF approaches for the detection of potential postural instability, exploiting time-based or DWT-based features, are comparable. As already mentioned, the added value in the use of DWT-based features could be more evident in case more challenging tasks, analyzing postural sway dynamics, have to be accomplished. This behavior confirms the outcomes of investigations presented in Section 4, highlighting the drawbacks of threshold-based algorithms related to the lack, for the observed set of features, of robust elements of separation between stable and unstable behaviors.

## 5. Concluding Remarks

This paper proposes a comparison among different techniques for the detection of the potential status of postural instability, exploiting a wearable sensing node, along with threshold-based algorithms or NF-based inference systems.

This task is strategic to allow for the continuous and real-time monitoring of the postural sway in elderly people and users affected by neurologic diseases, such as PD.

As demonstrated by the results shown in Table 3, the adopted algorithms perform very well in distinguishing among stable and unstable behaviors. Apparently, the performance of the three methodologies is also comparable in terms of reliability. However, the investigation developed in Section 4.4 demonstrated that NF-based strategies are characterized by better performance in terms of reliability, especially in the presence of noisy data.

Although not easy to implement in microcontroller-based architecture, NF inference systems perform better than the threshold-based algorithm. This being said, a convenient approach could be to validate the threshold-based real-time estimation of postural sway by implementing a data fusion strategy, which exploits the NF inference running off-line on a dedicated (cloud) server.

The presented analysis encourages further developments of the proposed strategies.

Further efforts will be dedicated to test the proposed methodologies by using a larger dataset and involving end-users. Moreover, the implementation of the NF detection paradigms into an embedded system will also be addressed, with the aim of performing a more reliable real-time detection of potential postural instability during the everyday life of frail people, while performing activities of daily living in their own premises.

## Figures and Tables

**Table 1:** The adopted dataset, including different postural movements (Standing (ST), Antero-Posterior (AP), Medio-Lateral (ML), Unstable (UNS)), and cases stated for node positions. Quantities indicate the number of patterns achieved under each condition.

	Case 1	Case 2	Case 3	Case 4	Case 5	Case 6
**ST**	47	50	58	58	53	51
**AP**	53	51	50	51	53	58
**ML**	52	48	52	52	47	50
**UNS**	49	52	50	50	52	52

**Table 2:** Main features adopted through this work to implement algorithms for the analysis of postural sway.

Feature	Description
$D_{AP}^{max}$$\;(m),\;D_{AP}^{min}$ (m)	Maximum and minimum displacement in the AP direction.
$D_{ML}^{max}$$\;(m),\;D_{ML}^{min}$ (m)	Maximum and minimum displacement in the ML direction.
$D_{RMS}=\sqrt{\frac{\sum_{1=1}^{N}{\left(d_{p}\left(i\right)\right)}^{2}}{N}}$ (m)	Root Mean Square (RMS) displacement. *dp(i)* is the distance between two adjacent points on the stabilogram.
$CEA_{95\%}=\pi *a*b$ (m^2^) $a=CSF*\sigma_{AP}$ (m) $b=CSF*\sigma_{ML}$ (m)	Ellipse area which includes 95% of the stabilogram plot. The two terms *a* and *b* represent the two semi-axes of the ellipse. CSF is a Confidence Scaling Factor whose value, in the case of the 95% ellipse, is 2.4477 [19,20]. *σ_AP_* and *σ_ML_* are the standard deviations of the *D_AP_* and *D_ML_*, respectively.

**Table 3:** Main features adopted through this work to implement algorithms for the analysis of postural sway.

	Q% Test Dataset	Q% Training Dataset	$RI_{Mean}^{}\%$ Test Dataset	$RI_{Mean}^{}\%$ Training Dataset	$RI_{Std}^{}\%$ Test Dataset	$RI_{Std}^{}\%$ Training Dataset
**Threshold Algorithm**	100	100	80.89	80.48 (*RI_JF_*) 100 (*RI_Th_*)	9.01 (*RI_JF_*) 0.00 (*RI_Th_*)	12.24 (*RI_JF_*) 0.00 (*RI_Th_*)
**Time-based Features and Neuro Fuzzy**	100	100	99.76	99.70	0.62	0.48
**DWT-based Features and Neuro Fuzzy**	100	100	99.09	98.59	0.75	3.31
